# Who Are the Flourishing Emerging Adults on the Urban East Coast of Australia?

**DOI:** 10.3390/ijerph18031125

**Published:** 2021-01-27

**Authors:** Ernesta Sofija, Neil Harris, Bernadette Sebar, Dung Phung

**Affiliations:** School of Medicine, Gold Coast Campus, Griffith University, Brisbane, QLD 4222, Australia; n.harris@griffith.edu.au (N.H.); b.sebar@griffith.edu.au (B.S.); d.phung@griffith.edu.au (D.P.)

**Keywords:** mental health, young adults, health promotion, predictors, social support

## Abstract

It is increasingly recognised that strategies to treat or prevent mental illness alone do not guarantee a mentally healthy population. Emerging adults have been identified as a particularly vulnerable population when it comes to mental health concerns. While mental illnesses are carefully monitored and researched, less is known about mental wellbeing or flourishing, that is, experience of both high hedonic and eudaimonic wellbeing. This cross-sectional study examined the prevalence of flourishing and its predictors among emerging adults in Australia. 1155 emerging adults aged 18–25 years completed a survey containing measures of wellbeing, social networks, social connectedness, health status, and socio-demographic variables. Most participants (60.4%) experienced moderate levels of wellbeing, 38.6% were flourishing and 1% were languishing (low wellbeing). Flourishers were more likely to be older, identify as Indigenous, be in a romantic relationship, study at university, perceive their family background as wealthy, rate their general health status as excellent, and have higher perceived social resources. The findings show that the majority of emerging adults are not experiencing flourishing and offer an insight into potential target groups and settings, such as vocational education colleges, for emerging adult mental health promotion. Interventions that help strengthen social resources have the potential to improve the mental wellbeing of emerging adults.

## 1. Introduction

Contemporary social and economic forces are making the transition into adulthood more complex than at any other point in history [1,2]. Consequently, emerging adulthood (18–29 years of age) has been conceived as a distinct developmental stage that young people go through as they transition into adulthood, particularly in highly industrialised societies [1,3]. While emerging adulthood is a positive experience for most, this stage of life is the most turbulent with rapid and frequent lifestyle changes, such as in living arrangements, jobs, study, and romantic relationships, which have been linked to increased stress and pressure on coping resources [1,2,4]. Simultaneously, global and national statistics increasingly identify this age group as the most vulnerable to mental health and behavioural challenges that translate into a significant disease burden [5,6,7,8,9].

The World Health Organization [10] defines mental health as “a state of well-being in which every individual realizes his or her own potential, can cope with the normal stresses of life, can work productively and fruitfully, and is able to make a contribution to her or his community” (p10). This definition implies that efforts to improve the mental health of populations should move beyond treatment to also focus on promoting wellbeing. Nevertheless, when it comes to improving mental health, the predominant focus is on the presence or absence of mental illness [11,12]. This is problematic, as it prevents a fuller understanding of mental health including the creation of more holistic interventions. It is increasingly recognised that only treating or preventing mental illness does not guarantee a mentally healthy population, as the absence of mental illness does not necessarily equate to mental health [13,14].

The current study draws on Keyes’ [13,15] two-continua model, in which mental health and mental illness are two separate but related dimensions of functioning. The mental illness dimension relates to the extent to which a disorder is present, while the mental health dimension concerns the presence of wellbeing. Individuals who exhibit high levels of hedonic (positive feelings) and eudaimonic (positive functioning) wellbeing are flourishers [13,15,16]. On the opposite end of the mental health continua are the languishers, and those in between are considered to experience moderate mental health. These states can coexist with the presence or absence of mental ailments, and the processes that impact mental health can differ from those that concern mental illness [12,16,17].

Flourishing is more than just a pleasant state. Flourishing mental health has been associated with a number of tangible benefits, such as reduced suicide risk [18], increased longevity [19,20,21], and reduced incidence of mental issues, such as depression and anxiety [22,23]. Keyes [13] found that completely mentally healthy (no mental illness and flourishing) individuals reported the fewest missed days or work cutbacks, lower risk of cardiovascular and many other chronic physical diseases linked with age, and lower health care utilisation. Among young people, flourishing has been linked to higher academic achievement [24] and reduced health risk behaviour [11,25]. Higher levels of wellbeing have been found to serve some protective function with an inverse association with wellbeing being strongest for more dangerous types of drug use, unsafe sexual behaviour, and reckless driving [26]. Thus, understanding and promoting a state of flourishing can be an important strategy to not only achieve optimal mental health of populations, but also to generate additional health and societal benefits.

Flourishing research is relatively new, with research on prevalence, predictors, and characteristics of flourishers tending to focus on hedonic or eudaimonic wellbeing [27,28,29]. The existing studies on flourishing suggest that the rates and predictors of flourishing vary across countries and populations [11,16,30,31]. For example, Schotanus-Dijkstra et al.’s [28] study revealed that 36.5% of Dutch people were flourishers, with the highest rates of flourishing (45.2%) observed in the youngest age group (18–24 years). In contrast, Keyes and Simoes [20] found the highest percentage of flourishers were 45- to 54-year-olds (22.6%) and the lowest rates were in the younger age groups (15.5%). While the results are mixed, some of the predictors of flourishing that have been identified in the literature include age, gender, level of education, employment, marital status and living arrangements, social support, health status, and personality traits [16,20,28,32]. In summary, the literature suggests that flourishing rates and its predictors can be unique to specific population groups and contexts.

How young people transition through emerging adulthood depends upon the interaction between their environment and personal, family and social resources [2]. To better understand how emerging adults are faring, and to support a healthy transition to adulthood, there is a need to know both the prevalence and predictors of mental ill-health and also of flourishing. To the best of our knowledge, there is no published research on the prevalence and predictors of flourishing in the emerging adult population of Australia. Thus, this study aims to examine the status of mental wellbeing of emerging adults on the urban east coast of Australia through the following research questions: (i) What is the prevalence of flourishing? (ii) What are the key demographic characteristics associated with flourishing? and (iii) How do social resources (social networks and social connectedness) and health status relate to emerging adult wellbeing?

## 2. Materials and Methods

### 2.1. Study Design, Participant Recruitment, and Data Collection

A cross-sectional quantitative survey was conducted with emerging adults who resided in South East Queensland and Northern New South Wales. A convenience sample was drawn using a multi-strategy approach combining online (using LimeSurvey, an online survey tool; LimeSurvey, Hamburg, Germany) and face-to-face (paper-pencil survey) data collection at various community locations. Prospective participants were approached through complementary means, such as online advertisements (e.g., Facebook), posters and flyers distributed across council libraries, coffee shops, shopping centres, workplaces, and similar locations where emerging adults are likely to congregate, as well as through a university research volunteers broadcast email. Furthermore, participants were directly approached at train stations and local colleges where they were provided with the paper-pencil survey or the link to the online survey. To ensure participant confidentiality, all data collected was de-identified with respondents not identifiable in any publication or reporting resulting from the research. 

### 2.2. Measures

#### 2.2.1. Mental Wellbeing

Wellbeing was measured using Keyes’ Mental Health Continuum—Long Form (MHC—LF) tool [13,15,20], which consists of 40 items across three subscales: emotional (EWB), social (SWB), and psychological (PWB) wellbeing. EWB measures relate to hedonic wellbeing (positive feelings), while PWB and SWB scales are measures of eudaimonic wellbeing (positive functioning). To assess EWB, participants were asked to report on the frequency of six positive emotions (e.g., feeling cheerful) during the previous 30 days on a five-point Likert scale, from none of the time to all the time. In addition, participants rated their overall satisfaction with life by rating a single item scale from 0 (worst you have ever been) to 10 (best you have ever been). The PWB scale includes six three-item subscales to measure six psychological wellbeing dimensions: self-acceptance, personal growth, positive relations with others, purpose in life, autonomy, and environmental mastery. The SWB scale measures five dimensions of social wellbeing through five three-item subscales including social acceptance, social coherence, social actualization, social contribution, and social integration. For these two scales, participants rate their wellbeing from strongly disagree to strongly agree. PWB and SWB dimensions represent the 11 diagnostic criteria of eudaimonic wellbeing, and the two measures of EWB represent two diagnostic criteria of hedonic wellbeing [15]. High internal consistency (Cronbach’s alpha) for each of the three measures has been reported (>0.80; see for example, Keyes (2005).

Based on the MHC—LF measure, wellbeing can be assessed as a continuous or categorical variable, with each approach offering additional insights [13,15]. Given that it is the characteristics of flourishers that are of interest in this study, the categorical assessment of wellbeing was used. The scores for each of the 13 diagnostic criteria were calculated, standardised, and computed into tertiles [15]. Dummy variables were created for diagnosis and subsequent analyses. Based on these criteria, participants were diagnosed as flourishing if they scored highly (top tertile) on either of the two scales of EWB, and on at least six of the 11 criteria of positive functioning [18]. Following the same diagnostic logic, participants who exhibited low levels (bottom tertile) on these criteria were categorized as languishers. Participants who did not meet the criteria for either flourishing or languishing were considered to experience moderate mental wellbeing [13,18].

#### 2.2.2. Socio-Demographic Factors

Socio-demographics included age, gender, ethnicity, place of birth, religion, marital/relationship status, parental status, living arrangements, level of education of both participants and their parents/guardians, current study and employment status, sources of financial support, personal income from all sources, and perceived family wealth.

#### 2.2.3. Social Resources and Health Status

Social networks that relate to perceived availability of social support [33] were measured using the Lubben Social Network six-item scale with three questions for each of the resources, namely family and friends. The questions refer to the number of people ranging from none to nine or more that individuals see at least once a month, feel at ease with that they can talk about private matters, and feel close to so that they can call on them for help. The total score was calculated by finding the sum of the items. Possible score ranges between 0–30, with a higher score indicating higher perceived social support. Originally this scale was developed for research with the elderly; however, this and very similar measures have been used with the general population including the emerging adult age group [28,34].

Social connectedness (SC), a psychological sense of belonging, or how individuals cognitively construe interpersonal closeness with others in their social world [32,33,35] was measured using the 15-item Social Connectedness Scale (α = 0.93) [35]. Individuals with a low level of SC feel less accepted, cannot get support from their environment, and may lack life goals or motivation to achieve these [36,37]. The scale uses a 6-point rating scale (1 = strongly disagree to 6 = strongly agree). The total SC scores were calculated following the guidelines and the possible score ranges between 15–90 [35].

Self-rated health status was measured using a standard one-item measure where participants rate their health in general as excellent, very good, good, fair or poor [38]. Body Mass Index (BMI) as an indicator of health was also measured [39]. BMI was calculated based on participant self-reported weight and height and computed into four internationally recognised BMI categories: (1) Underweight ≤18.49; (2) Normal weight 18.50–24.99; (3) Overweight 25.00–29.99, and (4) Obese BMI 30 ≥ [40]. While there has been criticism regarding the use of self-reported BMI, it has been shown to be a reliable and valid estimate of measured BMI in emerging adults [41].

### 2.3. Data Analysis

Data cleaning was undertaken to identify missing data and outliers and to eliminate data entry errors. Prevalence of wellbeing diagnoses was obtained with descriptive statistics. Due to a small number of languishers identified in our study, for further analysis, a dummy variable was created with two categories: (0) non-flourishers, languishers and moderately mentally well individuals, and (1) flourishers. We were also interested in examining and comparing the characteristics of individuals with high levels of eudaimonic and hedonic wellbeing. Therefore, for the hedonic and eudaimonic wellbeing variables, two binary categories were created: high (1) and not high (0) wellbeing. Descriptive statistics were undertaken for the socio-demographic factors, health status indicators, and social resources for flourishers versus non-flourishers, and differences were tested with Chi square statistics and *t*-tests. Then, all variables significantly associated with flourishing were included in a multivariate logistic regression (MLR) analysis using a forced entry method, in which all predictor variables were tested in one block to assess the relationships while controlling for the effects of other predictors. These steps were repeated with the binary variables of high and not high hedonic and eudaimonic wellbeing. Data analyses were performed using IBM SPSS 25.0.

## 3. Results

In total 1264 participants completed the survey with 109 cases excluded from further analysis during data cleaning. Thus, the final sample was 1155 emerging adults aged 18–25 years (Mean age: 20.67 years). Females comprised 74.8% (*n* = 857) of the sample. The majority of respondents were Caucasian (74.9%, *n* = 855) and were born in Australia (75.8%, *n* = 869); 80.9% (*n* = 929) of respondents were full or part-time university students, 12.4% (*n* = 142) reported studying at technical education institutions (technical and further education (TAFE)/vocational education and training (VET)) and 6.8% (*n* = 78) were not currently enrolled in any course.

Table 1 presents the descriptive statistics and Cronbach’s alpha for wellbeing and social resources variables. The means and observed ranges are similar to those reported in other studies [15,24].

Data analysis revealed that 38.6% (*n* = 444) of participants were flourishers and 61.4% were non-flourishers (1%, *n* = 11 languishers, 60.4%, *n* = 695 moderately mentally well). It also revealed that 58.2% (*n* = 672) of emerging adults exhibited high levels (top tertile) and 15.4% (*n* = 178) low levels (bottom tertile) of hedonic wellbeing, and respectively 47.1% (*n* = 544) and 1.4% (*n* = 16) for eudaimonic wellbeing. As with the flourishing variable, for subsequent analyses, we grouped and contrasted two groups of not high (41.5%, *n* = 477) versus high hedonic wellbeing, and not high (52.9%, *n* = 611) versus high eudaimonic wellbeing.

Table 2 and Table 3 present the results on the relationship between flourishing, high hedonic, and high eudaimonic wellbeing and key socio-demographic factors, health status, and social resources based on descriptive statistics and univariate analyses. Table 2 and Table 3 show that 10 out of 15 socio-demographic factors were significantly associated with flourishing. No statistically significant relationships were found between flourishing and gender, place of birth, living arrangements, parental education, and number of sources of financial support (*p* > 0.05). Flourishers were significantly more likely to rate their general health status as excellent (*p* < 0.001) and to be in the normal BMI weight category (*p* < 0.01). The mean scores of social network and social connectedness measures were also significantly (*p* < 0.001) higher among flourishing emerging adults.

Similar significant relationships as for flourishing were found between eudaimonic wellbeing and socio-demographics, health status, and social resources. However, no significant relationship was found between high and not high eudaimonic wellbeing groups and having children as well as BMI categories. In contrast, the findings around high and not high hedonic wellbeing groups varied more distinctly compared to flourishing, with a few more factors not reaching statistically significant differences. Factors that were statistically significantly related to flourishing but not to high hedonic wellbeing were ethnicity, religion, having children, current study status, and social connectedness. Living with a partner compared to other living arrangements was a significant factor linked to high hedonic and high eudaimonic wellbeing (*p* < 0.05) but was not significant for flourishing.

Table 4 presents the results of the MLRs, which were performed to examine the relationship between multiple factors and wellbeing (flourishing, hedonic, and eudaimonic wellbeing).

Fourteen factors were used as predictors for flourishing, both categorical and continuous (Table 4). As shown in Table 4, significant relationships were found for eight factors. Participants aged 18–19 years were 55% less likely to be flourishers compared to those aged 24–25 (OR, 0.45; 95% CI: 0.23–0.87). Participants who identified themselves as Asian were 74% (OR, 0.26; 95% CI: 0.11–0.61), and those who were not in a relationship were 42% (OR, 0.58; 95% CI: 0.4–0.85) less likely to be flourishers in comparison with Indigenous and Other (Other ethnicity group includes ethnicities that contained small participant numbers and therefore were grouped into one group for the analysis. Other group includes: mixed, Middle Eastern, African, and Hispanic.) ethnicity groups and those who reported to be in a relationship, respectively. Emerging adults who indicated currently studying part-time or full-time at TAFE/VET were 60% (OR, 0.4; 95% CI: 0.21–0.74) less likely to be flourishers compared to university students. In terms of perceived family wealth, all groups were 65–76% less likely to be considered flourishing compared to those who perceived their family background as wealthy (Table 4). Self-rated health status was found to be a highly significant predictor of flourishing with those who rated their health as poor being 97% (OR, 0.03; 95% CI: 0.00–0.29) less likely to flourish. Furthermore, a one unit increase in social connectedness and social networks resulted in 12% (OR, 1.12; 95% CI: 1.1–1.14) and 18% (OR, 1.18; 95% CI: 1.14–1.22) increase in flourishing, respectively.

For eudaimonic wellbeing significant relationships were found for eight out of 13 examined factors (Table 4). Similar significant relationships and magnitudes were observed as flourishing for most factors, except relationship status, which was not significantly associated with eudaimonic wellbeing but was significant for flourishing. Education was also significantly associated with high eudaimonic wellbeing but not with flourishing or high hedonic wellbeing (Table 4).

For hedonic wellbeing, significant relationships were found for six out of 10 examined factors (Table 4). Employment status was significantly associated with high hedonic wellbeing, which was not the case with flourishing or high eudaimonic wellbeing. Participants who were employed part-time and unemployed were 50% (OR, 0.50 95% CI: 0.26–0.99) and 53% (OR, 0.47; 95% CI: 0.23–0.97) less likely to experience high hedonic wellbeing, respectively, compared to those employed full-time.

## 4. Discussion

This research highlights the need to attend to mental wellbeing to improve overall health and wellbeing among emerging adults in the urban east coast of Australia. Only 38.6% (*n* = 444 of 1150) of emerging adults were flourishing, with the majority experiencing moderate mental health. While it is promising that very few participants (1%) were languishing, research suggests that individuals who experience moderate mental health compared to flourishers experience comparatively poorer outcomes across life domains such as health, health-related behaviour, and academic performance, that may negatively impact life trajectories [11,22]. The findings broaden our understanding of mental wellbeing and highlight the need for our theoretical models to encompass health as well as disease to inform practice.

The findings show that measures of both hedonic and eudaimonic wellbeing should be used to inform the development and evaluation of mental health promotion interventions. Based on our findings, 58.2% (*n* = 672 of 1149) of emerging adults exhibited high levels of hedonic wellbeing, and 47.1% (*n* = 544 of 1155) of eudaimonic wellbeing. This suggests that a larger proportion of emerging adults meet hedonic or eudaimonic wellbeing criteria of flourishing, but a substantially smaller (38.6%) proportion meet both criteria. It is common in population health surveys aimed to measure mental health to use the measures of mental illness (see for example [42]) or measures that capture only one of the set of symptoms of mental wellbeing, such as satisfaction with life. Our findings support the arguments that using such measures fail to capture more accurate assessment of mental health of populations [11,15].

Furthermore, by identifying characteristics of flourishers, this study sheds light on certain subgroups of emerging adults that could benefit from interventions. For example, TAFE/VET students were 60% less likely to be flourishers compared to university students, indicating that this population is at higher risk of lower mental wellbeing. Bonevski, Guillaumier, Paul, and Walsh [43] found that TAFE students in Australia had a high prevalence of unhealthy lifestyle behaviours and positioned vocational education organisations as a setting for health promotion. Further research is needed to understand why TAFE/VET students experience lower levels of mental wellbeing compared to university students. At a practical level, interventions to specifically target mental wellbeing of TAFE/VET students with a particular focus on strengthening eudaimonic wellbeing need to be developed.

The relationships between wellbeing variables and education, employment, personal annual income, and perceived family wealth were mixed. While education was associated with eudaimonic wellbeing, employment was associated only with hedonic wellbeing. Interestingly, personal annual income from all sources was not associated with wellbeing, although perceived family wealth was associated with all wellbeing variables. These findings represent the transitional nature of the population being studied. While the association between wellbeing and higher education is unclear, the findings from the current study suggest personal growth in completing higher education may contribute to emerging adult wellbeing [44]. The association between employment and hedonic wellbeing could be linked to the security and independence enjoyed through full time employment providing the basis for exploring opportunities for pleasure [45,46]. The significant relationship between wellbeing and perceived family wealth but not the actual personal annual income may indicate the importance of perceived social status and support from family in transition to adulthood [47,48].

The finding around the relationship between ethnicity and flourishing and high eudaimonic wellbeing, in particular, Indigenous and Other ethnicity groups (*n* = 123) experiencing higher wellbeing compared to those who identified as Caucasians and Asians, was unexpected. While the percentage of Indigenous participants in our study was relatively low 4.01% (*n* = 47), such a proportion is consistent with national census statistics [49]. Considerable disparities in health between Indigenous and non-Indigenous populations are regularly documented with Indigenous populations experiencing poorer health, including the highest rates of mental illness [50]. This has been linked to several determinants of health, such as social disadvantage, colonisation, and the destruction of Indigenous culture [50]. Keyes [51] reported a Black–White Paradox in health in his study where he found that Blacks had higher rates of flourishing than Whites and were more mentally resilient in the face of greater social inequality, exposure to discrimination, and high rates of physical morbidity. It is plausible that the higher flourishing rates among Indigenous and Other ethnicity groups of emerging adults in our study could be linked to resilience, or in fact, point to the strength among these sub-populations that could be built upon in health promotion interventions, which is often overlooked in traditional deficit-focused approaches [50,51,52]. Given the modest sample of Indigenous and Other ethnicity groups, future research should aim to oversample these populations to improve our understanding of the determinants of their wellbeing.

In contrast, Asians were less likely to experience flourishing and high eudaimonic wellbeing compared to all other ethnic (including Caucasian) groups. Lower self-reported levels of wellbeing among Asians has been linked to culture (individualistic vs. collectivistic), lower felt understanding, and potential cultural and language biases when answering the survey questions [53,54,55]. Due to the latter, it is advised to interpret with caution the ethnic differences in survey data on wellbeing and similar constructs [53]. Nevertheless, our findings point to the need to consider cultural differences when designing interventions to promote flourishing in emerging adults, particularly in multi-cultural countries such as Australia [55].

Furthermore, our findings indicate that flourishers were more likely to have access to social resources, and thus mental health promotion interventions should take this into consideration to help build both social networks and social connectedness among emerging adults to promote flourishing. This is consistent with previous research [28,32,33]. Eraslan-Capan [32] found that university students with low levels of social connectedness were more likely to be pessimistic regarding the future including their ability to change it, which resulted in low flourishing. Emerging adults are adjusting to new environments (e.g., commencing tertiary studies), and some to changes in social resources, such as diminishing social support due to moving away from the family home [1,56]. This may also explain why our youngest participants, who have just commenced their transition, had lower rates of flourishing. Fink [30] found that supportive college environments, sense of belonging, and civic engagement were some of the predictors of flourishing among students. This illustrates that tertiary education institutions can be an appropriate setting for mental wellbeing promotion in a subgroup of emerging adults. Overall, this study highlights the importance of moving beyond mental illness to more fully understand and support mental wellbeing in emerging adulthood.

This study has several limitations. The cross-sectional design makes it difficult to draw conclusions about directionality of associations, and despite the efforts to diversify the sample, it consisted of predominantly Caucasian females and university students. Overrepresentation of these populations is an ongoing issue in social science and psychology research, which limits the generalisability of findings [57,58]. The present study did manage to recruit a reasonable number of males, TAFE/VET students and non-Caucasian participants, which offered insights into their wellbeing. Nevertheless, some caution is needed when generalising the results to the broader emerging adult population. Furthermore, limitations relate to the issues associated with the use of self-reported measures, which can be subject to individual interpretation and misreporting, such as offering socially desirable responses [59]. That said, the scales used to measure key study variables have been successfully used in research across various populations, which lessens concerns associated with the use of self-reported measures.

## 5. Conclusions

The findings of this study reveal that most emerging adults on the urban east coast of Australia do not experience flourishing mental health. At a theoretical level, the findings emphasise the importance of broadening our understanding of mental health to acknowledge that the absence of mental illness does not equate with mental health. At a practical level, the findings highlight the importance of focusing health promotion efforts on encouraging mental wellbeing and addressing the factors that lower it, while simultaneously treating mental ailments to achieve optimal mental health in this population subgroup. Interventions that help build or strengthen social resources among emerging adults have the potential to improve mental health in young people in their transition to adulthood. Further research is needed to understand how and what other resources emerging adults utilise and how to best facilitate these with health promotion efforts to promote and sustain flourishing mental health.

## Figures and Tables

**Table 1:** Descriptive statistics for wellbeing and social resources variables.

Variable	N	M	SD	Observed Range	Possible Range	α
EWB *	973	9.77	2.65	1.5–15	1–15	0.90
PWB **	1119	26.85	3.83	14.33–36	6–36	0.83
SWB **	1111	19.63	3.47	8–30	5–30	0.82
Social Network	1140	17.58	5.325	0–30	0–30	0.81
Social Connectedness	1117	63.14	14.716	17–90	15–90	0.94

* Emotional wellbeing (EWB) overall score was calculated by averaging six items and adding the single item of overall life rating. ** Overall scores for psychological wellbeing (PWB) and social wellbeing (SWB) scales were calculated by summing the items and dividing by three.

**Table 2:** Descriptive statistics and univariate analysis of wellbeing queried by socio-demographic factors and health status.

	Wellbeing Total			Hedonic Wellbeing			Eudaimonic Wellbeing		
	Non-Flourishers	Flourishers		Not High	High		Not High	High	
*n* (%)	706 (61.4)	444 (38.6)		477 (41.5)	672 (58.5)		611 (52.9)	544 (47.1)	
	*n* (%)	*n* (%)	*p*	*n* (%)	*n* (%)	*p*	*n* (%)	*n* (%)	*p*
Age			*p* < 0.001			*p* < 0.01			*p* < 0.001
18–19	309 (68.4)	143 (31.6)		207 (46.1)	242 (53.9)		274 (60.4)	180 (39.6)	
20–21	183 (62)	112 (38)		126 (42.6)	170 (57.4)		154 (52)	142 (48)	
22–23	128 (55.4)	103 (44.6)		90 (38.8)	142 (61.2)		110 (47.4)	122 (52.6)	
24–25	85 (50)	85 (50)		53 (31.2)	117 (68.8)		72 (42.1)	99 (57.9)	
Gender			*p* > 0.05			*p* > 0.05			*p* > 0.05
Male	165 (57.7)	121 (42.3)		106 (37.1)	180 (62.9)		153 (52.9)	136 (47.1)	
Female	535 (62.6)	320 (37.4)		367 (43)	487 (57)		452 (52.7)	405 (47.3)	
Ethnicity			*p* < 0.01			*p* > 0.05			*p* < 0.001
Caucasian	509 (59.5)	346 (40.5)		348 (40.7)	506 (59.3)		438 (51.2)	417 (48.8)	
Asian	120 (75)	40 (25)		72 (45.3)	87 (54.7)		109 (67.3)	53 (32.7)	
Indigenous ^1^	26 (55.3)	21 (44.7)		17 (37)	29 (63)		21 (44.7)	26 (55.3)	
Other ^1^	42 (55.3)	34 (44.7)		31 (40.8)	45 (59.2)		34 (44.2)	43 (55.8)	
Place of birth			*p* > 0.05			*p* > 0.05			*p* > 0.05
Australia	543 (62.7)	323 (37.3)		370 (42.7)	497 (57.3)		470 (54.1)	399 (45.9)	
Overseas	159 (57.6)	117 (42.4)		102 (37.4)	171 (62.6)		137 (49.5)	140 (50.5)	
Religion			*p* < 0.05			*p* > 0.05			*p* < 0.05
No	442 (63.9)	250 (36.1)		301 (43.4)	392 (56.6)		386 (55.5)	309 (44.5)	
Yes	240 (56.7)	183 (43.3)		164 (39)	257 (61)		204 (48)	221 (52)	
Romantic relationship status ^2^			*p* < 0.001			*p* < 0.001			*p* < 0.001
Not in relationship	447 (65.8)	232 (34.2)		313 (46.2)	365 (53.8)		389 (57)	293 (43)	
In relationship	248 (54.3)	441(45.7)		157 (34.4)	300 (65.6)		213 (46.4)	246 (53.6)	
Children			*p* < 0.05			*p* > 0.05			*p* > 0.05
No	693 (61.6)	432 (38.4)		470 (41.8)	654 (58.2)		598 (52.9)	532 (47.1)	
Yes	5 (33.3)	10 (66.7)		4 (26.7)	11 (73.3)		5 (33.3)	10 (66.7)	
Living arrangements			*p* > 0.05			*p* < 0.05			*p* < 0.05
Alone	28 (66.7)	14 (33.3)		24 (57.1)	18 (42.9)		26 (61.9)	16 (38.1)	
Parents and/or other family	359 (64.2)	200 (35.8)		247 (44.1)	313 (55.9)		317 (56.3)	246 (43.7)	
Partner	99 (55.9)	78 (44.1)		62 (34.8)	116 (65.2)		83 (46.6)	95 (53.4)	
Friends/housemates	212 (58.4)	151 (41.6)		139 (38.6)	221 (61.4)		178 (49)	185 (51)	
Education			*p* < 0.001			*p* < 0.05			*p* < 0.001
Finished year 12 or less	444 (64.4)	245 (35.6)		301 (43.9)	385 (56.1)		390 (56.4)	301 (43.6)	
Diploma/certificate/trade	141 (65)	76 (35)		95 (43.4)	124 (56.6)		122 (55.7)	97 (44.3)	
University degree ^3^	116 (48.7)	122 (51.3)		79 (33.2)	159 (66.8)		94 (39.3)	145 (60.7)	
Education of either of parent/guardian			*p* > 0.05			*p* > 0.05			*p* > 0.05
Finished year 12 or less	217 (63.1)	127 (36.9)		133 (38.9)	209 (61.1)		192 (55.7)	153 (44.3)	
Diploma/certificate/trade	114 (56.4)	88 (43.6)		89 (44.1)	113 (55.9)		94 (46.3)	109 (53.7)	
University degree ^3^	354 (61)	221 (39)		236 (41.6)	331 (58.4)		298 (52.5)	270 (47.5)	
Other or Don’t know	21 (77.8)	6 (22.2)		13 (48.1)	14 (51.9)		18 (64.3)	10 (35.7)	
Current study status			*p* < 0.01			*p* > 0.05			*p* < 0.01
Full-time/part-time University	549 (59.3)	377 (40.7)		375 (40.5)	552 (59.5)		471 (50.7)	458 (49.3)	
Full-time/part-time TAFE/VET ^4^	103 (73.6)	37 (26.4)		70 (50)	70 (50)		95 (66.9)	47 (33.1)	
Not studying	49 (62.8)	29 (37.2)		29 (38.2)	47 (61.8)		40 (51.3)	38 (48.7)	
Employment			*p* < 0.001			*p* < 0.001			*p* < 0.01
Full-time ^5^	57 (54.8)	47 (45.2)		28 (27.2)	75 (72.8)		47 (45.2)	57 (54.8)	
Part-time ^5^	336 (56.7)	257 (43.3)		225 (38)	367 (62)		289 (48.7)	305 (51.3)	
Unemployed	293 (69.4)	129 (30.6)		211 (49.9)	212 (50.1)		256 (60.1)	170 (39.9)	
Other ^6^	9 (47.4)	10 (52.6)		6 (31.6)	13 (68.4)		9 (47.4)	10 (52.6)	
Personal annual income (all sources)			*p* < 0.05			*p* < 0.05			*p* < 0.05
No income	105 (71.4)	42 (28.6)		75 (50.7)	73 (49.3)		94 (63.5)	54 (36.5)	
$1–$12,999	246 (60.6)	160 (39.4)		171 (42.1)	235 (57.9)		209 (51.4)	198 (48.6)	
$13,000–$31,199	220 (58.8)	154 (41.2)		137 (36.6)	237 (63.4)		191 (50.9)	184 (49.1)	
$31,200 or more	56 (53.8)	48 (46.2)		36 (35)	67 (65)		45 (43.3)	59 (56.7)	
Perceived family wealth ^7^			*p* < 0.01			*p* < 0.01			*p* < 0.01
Wealthy	26 (41.9)	36 (58.1)		17 (27.4)	45 (72.6)		22 (35.5)	40 (64.5)	
Quite well-off	427 (60.7)	276 (39.3)		279 (39.7)	424 (60.3)		368 (52.1)	339 (47.9)	
Not very well-off	202 (64.5)	111 (35.5)		146 (46.8)	166 (53.2)		174 (55.4)	140 (44.6)	
Quite poor	40 (76.9)	12 (23.1)		29 (55.8)	23 (44.2)		36 (69.2)	16 (30.8)	
Sources of personal financial support ^8^			*p* > 0.05			*p* > 0.05			*p* > 0.05
One source	321(65.5)	169 (34.5)		218 (44.5)	272 (55.5)		283 (57.4)	210 (42.6)	
Two sources	211 (59.6)	143 (40.4)		138 (39.1)	215 (60.9)		181 (51)	174 (49)	
Three sources	106 (57.9)	77 (42.1)		75 (41)	108 (59)		88 (47.8)	96 (52.2)	
Four or more	40 (55.6)	32 (44.4)		28 (38.9)	44 (61.1)		34 (47.2)	38 (52.8)	
Body mass index (BMI)			*p* < 0.01			*p* < 0.01			*p* > 0.05
Underweight	84 (66.1)	43 (33.9)		51 (40.8)	74 (59.2)		73 (57)	55 (43)	
Healthy weight	431 (58.2)	309 (41.8)		291 (39.2)	451 (60.8)		376 (50.5)	368 (49.5)	
Overweight	111 (62)	68 (39)		72 (40.2)	107 (59.8)		97 (54.2)	82 (45.8)	
Obese	55 (82.1)	12 (17.9)		43 (65.2)	23 (34.8)		44 (65.7)	23 (34.3)	
Self-rated health status			*p* < 0.001			*p* < 0.001			*p* < 0.001
Excellent	37 (35.9)	66 (64.1)		22 (21.2)	82 (78.8)		30 (28.8)	74 (71.2)	
Very good	165 (47.6)	182 (52.4)		97 (28)	249 (72)		142 (40.9)	205 (59.1)	
Good	312 (65.8)	162 (34.2)		216 (45.5)	259 (54.5)		265 (55.7)	211 (44.3)	
Fair	156 (83.9)	30 (16.1)		112 (60.9)	72 (39.1)		142 (75.5)	46 (24.5)	
Poor	30 (93.8)	2 (6.3)		25 (78.1)	7 (21.9)		26 (81.3)	6 (18.8)	

^1^ Indigenous includes Aboriginal and/or Torres Strait Islander, Pacific Islander; Other includes mixed, Middle Eastern, African, Hispanic.; ^2^ Not in a relationship group includes single, unattached (not committed/casual) and divorced/separated; In a relationship, includes ongoing relationship, married, de-facto; ^3^ Undergraduate or Postgraduate; ^4^ TAFE/VET—technical and further education/vocational education and training; ^5^ Includes permanent, contract, casual; ^6^ Includes very sporadic casual work, holiday work, seasonal and similar; ^7^ Wealthy—within the highest 25% in your country in terms of wealth; Quite well-off—within the 51–75% range; Not very well-off within 26–50% range; Quite poor—within the lowest 25%; ^8^ Participants could tick all that apply from the following sources of personal financial support: part-time/full time work; direct support from family, repayable loans from family; a private loan (bank, credit card, university); youth allowance; scholarship or bursaries; personal savings; other. For analysis, responses were then aggregated and recoded to reflect the number of sources of financial support.

**Table 3:** Descriptive statistics and univariate analysis of wellbeing queried by social resources.

	Wellbeing Total				Hedonic Wellbeing				Eudaimonic Wellbeing			
	Non-Flourishers	Flourishers			Not High	High			Not High	High		
	M; SD	M; SD	T-Test (df); SE Difference	*p*	M; SD	M; SD	T-Test; df; SE Difference	*p*	M; SD	M; SD	T-Test; df; SE Difference	*p*
Social networks ^1^	16.03; 5.23	20.08; 4.40	−13.50 (1135); 0.300	*p* < 0.001	15.51; 5.36	19.04; 4.79	11.65 (1132); 0.303	*p* < 0.01	15.50; 5.13	19.92; 4.51	15.36 (1138); 0.288	*p* < 0.01
Social connectedness ^1^	56.6; 13.06	73.47; 10.75	−22.38 (1114); 0.650	*p* < 0.001	55.35;	68.57;	16.38 (1110); 0.807	*p* > 0.05	54.92; 12.46	72.25; 11.26	24.29 (1115); 0.713	*p* < 0.01
					13.58	12.99						

^1^ Differences between the groups were tested with T-test.

**Table 4:** Multivariable logistic regression in examining the relationship between study factors and flourishing (*n* = 914), high hedonic (*n* = 955) and high eudaimonic wellbeing (*n* = 928).

	Flourishing	High Hedonic Wellbeing	High Eudaimonic Eellbeing
**Sociodemographic factors**	OR ^1^ (95% CI ^2^)	OR (95% CI)	OR (95% CI)
Age			
24–25	Referent ^3^	Referent	Referent
22–23	0.72 (0.39–1.35)	0.59 (0.35–0.98) *	0.79 (0.42–1.49)
20–21	0.61 (0.32–1.17)	0.65 (0.39–1.09)	0.79 (0.41–1.52)
18–19	0.45 (0.23–0.87) *	0.70 (0.41–1.19)	0.45 (0.23–0.88) *
Ethnicity			
Indigenous and Other ^4^	Referent	Not included in the model	Referent
Caucasian	0.58 (0.31–1.07)		0.45 (0.24–0.84) *
Asian	0.26 (0.11–0.61) **		0.18 (0.08–0.41) ***
Religion		Not included in the model	
Yes	Referent		Referent
No	0.80 (0.55–1.18)		0.78 (0.53–1.14)
Romantic relationship status			
In relationship	Referent	Referent	Referent
Not in relationship	0.58 (0.40–0.85) **	0.59 (0.42– 0.83) **	0.69 (0.45–1.06)
Children		Not included in the model	Not included in the model
Yes	Referent		
No	0.19 (0.02–1.69)		
Education			
University degree ^5^	Referent	Referent	Referent
Finished year 12 or less	0.63 (0.38–1.06)	0.90 (0.60–1.4)	0.54 (0.32–0.92) *
Diploma/certificate/trade certificate	0.82 (0.45–1.51)	0.86 (0.53–1.40)	0.76 (0.42–1.39)
Current study status		Not included in the model	
Full-time/part-time University	Referent		Referent
Not studying	0.59 (0.26–1.36)		0.81 (0.36–1.82)
Full-time/part-time TAFE/VET ^6^	0.40 (0.21–0.74) **		0.32 (0.17–0.58) ***
Employment			
Full-time ^7^	Referent	Referent	Referent
Part-time ^7^	0.74 (0.34–1.61)	0.50 (0.26-0.99) *	0.80 (0.37–1.74)
Unemployed	0.58 (0.24–1.36)	0.47 (0.23–0.97) *	0.71 (0.30–1.67)
Other	1.03 (0.22–4.74)	0.75 (0.21–2.63)	0.76 (0.18–3.32)
Personal annual income from all sources			
$31,200 or more	Referent	Referent	Referent
$13,000–$31,199	1.12 (0.51–2.48)	1.57 (0.82–3.01)	1.23 (0.55–2.74)
$1–$12,999	1.85 (0.80–4.28)	1.46 (0.74–2.88)	2.25 (0.96–5.25)
No income	1.67 (0.60–4.67)	1.21 (0.54–2.74)	1.59 (0.57–4.42)
Perceived family wealth			
Wealthy	Referent	Referent	Referent
Quite well-off	0.26 (0.11–0.61) **	0.44 (0.20–0.97) *	0.23 (0.09–0.59) **
Not very well-off	0.35 (0.15–0.86) *	0.38 (0.17–0.87) *	0.33 (0.12–0.87) *
Quite poor	0.24 (0.068–0.838) *	0.30 (0.11–0.82) *	0.21 (0.06–0.76) *
Living arrangements	Not included in the model		
Partner		Referent	Referent
Friends/housemates		0.89 (0.53–1.49)	0.85 (0.46–1.57)
Parents and/or other family		0.86 (0.52–1.42)	0.79 (0.43–1.44)
Alone		0.64 (0.27–1.51)	1.22 (0.42–3.58)
**Health indicators and social resources**	OR ^a^ (95% CI)	OR ^a^ (95% CI)	OR ^a^ (95% CI)
BMI			Not included in the model
Normal weight	Referent	Referent	
Overweight	1.06 (0.64–1.75)	1.14 (0.76–1.70)	
Underweight	0.78 (0.42–1.43)	1.02 (0.63–1.64)	
Obese	0.49 (0.20–1.22)	0.66 (0.34–1.27)	
Self-rated health status			
Excellent	Referent	Referent	Referent
Very good	0.52 (0.26–1.06)	0.60 (0.32–1.13)	0.41 (0.19–0.87) *
Good	0.22 (0.11–0.44) ***	0.29 (0.16–0.54) ***	0.22 (0.11–0.47) ***
Fair	0.12 (0.05–0.28) ***	0.19 (0.10–0.37) ***	0.13 (0.06–0.29) ***
Poor	0.03 (0.00–0.29) **	0.09 (0.03–0.28) ***	0.09 (0.02–0.41) **
Social connectedness	1.12 (1.10–1.14) ***	Not included in the model	1.14 (1.12–1.16) ***
Social networks ^8^	1.18 (1.14–1.22) ***	1.13 (1.09–1.16) ***	1.21 (1.17–1.25) ***

^1^ OR—odds ratio; ^2^ CI—confidence interval; ^3^ Referent refers to the reference level or category used to measure the relative effect of the study factors; ^4^ Indigenous include Aboriginal and/or Torres Strait Islander, Pacific Islander and Other includes mixed, Middle Eastern, African, Hispanic; ^5^ Undergraduate or Postgraduate; ^6^ TAFE/VET—technical and further education/vocational education and training; ^7^ Includes permanent, contract, casual; ^8^ Due to high correlation between social connectedness and social networks (based on Pearson’s correlation coefficient) logistic regression was performed separately for social networks in the flourishing and eudaimonic wellbeing analyses; * *p* < 0.05; ** *p* < 0.01; *** *p* < 0.001.

## Data Availability

The de-identified data presented in this study are available on reasonable request from the corresponding author. The data are not publicly available due to ethical considerations.
